# Cardiac Tamponade Secondary to Streptococcus pneumoniae-Associated Purulent Pericardial Effusion in an Immunocompetent Young Adult With Heavy Tobacco Exposure

**DOI:** 10.7759/cureus.117607

**Published:** 2026-10-07

**Authors:** Priya Ramcharan, Matthew Raghunanan, Arun R Katwaroo, Valmiki K Seecheran, Rajeev V Seecheran, Joel D Teelucksingh, Ian Ramnarine, Naveen A Seecheran

**Affiliations:** 1 Cardiology, Eric Williams Medical Sciences Complex, Champ Fleurs, TTO; 2 Thoracic Surgery, Eric Williams Medical Sciences Complex, Champ Fleurs, TTO; 3 Interventional Cardiology, Westshore Medical Complex, Port of Spain, TTO; 4 Interventional Cardiology, Trinidad Institute of Medical Technology, St. Augustine, TTO; 5 Internal Medicine, St. James Medical Complex, Port of Spain, TTO; 6 Internal Medicine, Eric Williams Medical Sciences Complex, Champ Fleurs, TTO; 7 Internal Medicine, University of Kansas Medical Center, Kansas City, USA; 8 Internal Medicine, San Fernando General Hospital, San Fernando, TTO

**Keywords:** cardiac tamponade (ct), pericardial window, pericardiocentesis, purulent pericardial effusion (ppe), streptococcus pneumoniae, video-assisted thoracoscopic surgery (vats)

## Abstract

We describe a case of a 41-year-old immunocompetent Caribbean South Asian man with a 25-pack-year smoking history who presented with cardiac tamponade secondary to a *Streptococcus* species-associated purulent pericardial effusion (PPE), with clinical and microbiological findings suggestive of *Streptococcus pneumoniae*. The patient underwent emergent pericardiocentesis with drainage of 600 mL of frank purulent fluid, followed by video-assisted thoracoscopic surgery with pericardial window and washout. He progressed from obstructive to septic shock and was successfully managed with combined surgical drainage, broad-spectrum antimicrobial therapy, and adjunctive colchicine. The clinician should maintain a high index of suspicion for PPE as a cause of cardiac tamponade, even in the immunocompetent host, and consider invasive pneumococcal disease as a potential etiology in patients with heavy tobacco exposure.

## Introduction

Purulent pericarditis (PP) is a rare but fulminant infection of the pericardial space, defined by macroscopic or microscopic pus within the pericardial sac [1,2]. In the pre-antibiotic era, PP complicated up to 40% of pneumococcal pneumonia [3]. However, with the widespread availability of antimicrobials and pneumococcal vaccines, the incidence has declined to fewer than one case per 18,000 hospital admissions [1,4]. Despite this contemporary rarity, PP remains universally fatal if untreated and continues to carry a mortality rate of 40% even with optimal modern management, largely due to its propensity for rapid progression to cardiac tamponade, septic shock, and constrictive pericarditis [5-7].

Approximately 40-50% of PP cases are attributable to Gram-positive bacteria, with *Streptococcus pneumoniae* being the most common when there is direct intrathoracic spread. In contrast, *Staphylococcus aureus* predominates in hematogenous and post-surgical contexts [1,2,8,9]. The principal pathogenic routes include contiguous spread from a pulmonary or pleural focus, hematogenous spread from a distant nidus, and direct extension from a subdiaphragmatic abscess [1,2].

Given the paucity of reported cases of *Streptococcus pneumoniae*-associated PP in the immunocompetent adult, particularly within the Caribbean diaspora, we describe a case of a 41-year-old immunocompetent Caribbean South Asian man with no known medical illnesses and a heavy lifetime tobacco burden who presented with cardiac tamponade secondary to a *Streptococcus* species-associated purulent pericardial effusion (PPE), with clinical and microbiological findings suggestive of *Streptococcus pneumoniae*. The patient underwent emergent pericardiocentesis, followed by video-assisted thoracoscopic surgery (VATS)-guided pericardial window with washout and two weeks of targeted antimicrobial therapy, with eventual full clinical recovery.

## Case presentation

A 41-year-old immunocompetent Caribbean South Asian man with a 25-pack-year smoking history presented to the emergency department with a three-day history of subjective fevers, intractable emesis, and central, dull, intermittent chest pain, rated 7/10 in severity. He denied any preceding upper respiratory tract symptoms, illicit or recreational drug use, recent travel, sick contacts, toxin or animal exposures, and his family history was non-contributory.

On physical examination, he was afebrile, tachycardic at 130-140 beats per minute, and hypotensive at 80-90 mmHg, requiring vasopressor support with norepinephrine (2 mcg/kg/min). The cardiovascular examination showed Beck’s triad: distended jugular veins, muffled heart sounds, and hypotension [10]. The respiratory and abdominal examinations were otherwise unremarkable. A 12-lead ECG demonstrated diffuse ST-segment elevation in leads II, III, aVF, and V1-V6, with PR depression, consistent with acute pericarditis (Figure 1A) [11].

**Figure 1:**
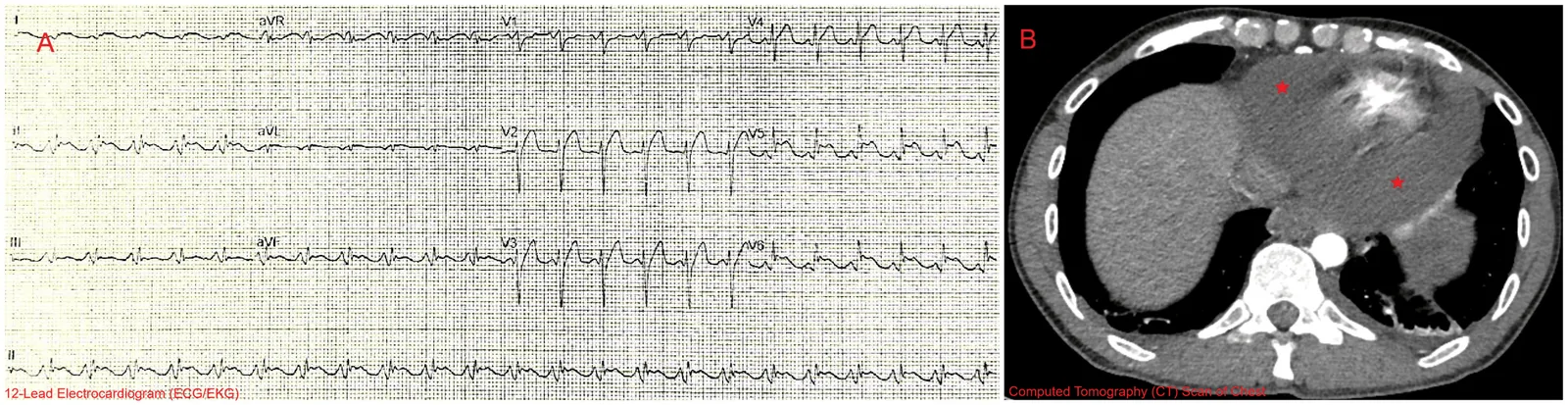
Patient's (A) 12-lead ECG showing diffuse ST-segment elevation in leads II, III, aVF, and V1-V6, with PR-segment depressions, consistent with acute pericarditis, and (B) cross-sectional CT image of the thorax showing a massive circumferential pericardial effusion (red asterisks) ECG: electrocardiogram, CT: computed tomography

Laboratory investigations were notable for a markedly elevated white cell count of 33.8 x 109/L (normal 4.0-10.3), C-reactive protein (CRP) of 462.9 mg/L (normal 0.0-10.0), an acute kidney injury with serum creatinine of 3.6 mg/dL (normal 0.7-1.2) and blood urea nitrogen of 37.2 mg/dL (normal 6.0-23.0), an elevated N-terminal pro-B-type natriuretic peptide of 18,146 pg/mL (normal <100), an elevated creatine kinase of 1702.3 U/L (normal 0.0-171.0) with a creatine kinase-myocardial band fraction of 39.5 U/L (normal 0.0-16.0), a normal high-sensitivity cardiac troponin of 0.01 ng/mL (normal 0.0-0.08), and a non-reactive human immunodeficiency virus (HIV) and COVID-19 screen (Table 1). A septic screen was also performed, with blood and urine cultures obtained. Contrast-enhanced chest CT showed a large pericardial effusion with trace bilateral pleural effusions and no consolidative, cavitary, or mediastinal abnormality (Figure 1B). A bedside two-dimensional transthoracic echocardiogram performed by the on-call cardiology team in the emergency department confirmed a large circumferential pericardial effusion with tamponade physiology, comprising right atrial systolic collapse, right ventricular diastolic collapse, and a plethoric, non-collapsing inferior vena cava [10,12].

**Table 1:** Patient's laboratory investigations SARS-CoV-2: severe acute respiratory syndrome coronavirus 2, IgG: immunoglobulin G, IgM: immunoglobulin M

Hematology	Values	Units	Reference value
Complete blood count			
White blood cells	33.8	10^3uL	4.0-10.3
Neutrophils	96.22	%	41.4-73.0
Lymphocytes	1.45	%	19.4-44.9
Monocytes	1.7	%	5.10-10.9
Eosinophils	0.51	%	0.50-6.00
Basophils	0.12	%	0.30-1.50
Neutrophils	32.52	10^3uL	2.10-6.10
Monocytes	0.57	10^3uL	0.00-0.20
Lymphocytes	0.49	10^3uL	1.30-3.50
Eosinophils	0.17	10^3uL	0.0-0.5
Basophils	0.04	10^3uL	0.00-0.20
Red blood cells	5.12	10^6uL	4.38-5.77
Hemoglobin	16.03	g/dL	13.6-17.2
Hematocrit	47.6	%	39.5-50.30
Mean corpuscular volume	93	fL	80.70-95.50
Mean corpuscular hemoglobin	31.3	pg	27.2-33.50
Mean corpuscular hemoglobin concentration	33.7	g/dL	32.7-35.6
Red cell distribution width	15.1	%	11.8-14.3
Platelet	274.2	10^3uL	159-388
Mean platelet volume	8.33	fL	6.90-10.8
Erythrocyte sedimentation rate	>120	mm/hr	0-10
Comprehensive metabolic panel			
Sodium	139	mmol/L	135-145
Potassium	4.1	mmol/L	3.5-5.1
Creatinine	3.6	mg/dL	0.7-1.2
Urea	37.23	mg/dL	6.0-23.0
Random blood glucose	106	mg/dL	<140
Calcium	8.99	mg/dL	8.6-10.2
Aspartate aminotransferase	90.47	IU/L	5.0-40.0
Alanine transaminase	40.11	IU/L	5.0-41.0
Alkaline phosphatase	76.13	U/L	40-129
Gamma-glutamyl transferase	45.81	U/L	6.0-61.0
Total bilirubin	2.85	mg/dL	0.0-1.29
Direct bilirubin	1.68	mg/dL	0.0-0.4
Indirect bilirubin	1.18	mg/dL	0.0-1.0
Total protein	6.44	g/dL	6.4-8.3
Albumin	3.61	g/dL	3.5-5.2
Globulin	2.83	g/dL	2.3-4.5
Magnesium	1.98	mg/dL	1.6-2.4
Phosphorus	1.65	mg/dL	2.5-4.5
Pericardial fluid analysis			
Glucose	36	mg/dL	40-70
Total protein	2	g/dL	6.3-8.6
Albumin	1	g/dL	3.5-5.6
Lactate dehydrogenase	298	U/L	120-246
Cholesterol	50	mg/dL	170-200
Triglycerides	41	mg/dL	40-150
Direct high-density lipoprotein	6	mg/dL	40-60
Very low-density lipoprotein	8	mg/dL	2.0-32.0
Low-density lipoprotein	36	mg/dL	60-130
Pericardial fluid acid-fast bacilli test	Negative	-	-
Pericardial tissue tuberculosis polymerase chain reaction	Negative	-	-
Cardiac pack			
Amylase	33.14	U/L	28-100
Lipase	18.36	U/L	10-140
C-reactive protein	462.88	mg/L	0.0-10.0
Lactate dehydrogenase	332.42	U/L	135-225
High-sensitivity cardiac troponin I	0.01	ng/dL	0.0-0.08
Creatine kinase	1702.28	U/L	0.0-171.0
Creatine kinase myocardial band	39.52	U/L	0.0-16.0
D-dimer	0.4	ng/mL	<0.5
B-type natriuretic peptide	18146	pg/mL	<100
Cholesterol	178	mg/dL	170-200
Triglycerides	98	mg/dL	40-150
Direct high-density lipoprotein	54	mg/dL	40-60
Very low-density lipoprotein	14	mg/dL	2.0-32.0
Low-density lipoprotein	100	mg/dL	60-130
Glycosylated hemoglobin	4.8	%	<5.7
HIV I and II antibodies	Negative	-	Negative
Mantoux	0	mm	0-5
SARS-CoV-2 antigen	Negative	-	Negative
Leptospira IgG	Negative	-	Negative
Leptospira IgM	Negative	-	Negative

An emergent ultrasound-guided pericardiocentesis was performed at the bedside following a stat dose of intravenous ceftriaxone 2 g, with drainage of approximately 600 mL of frank purulent fluid (Figure 2). Although hemodynamics improved transiently, the clinical picture evolved from obstructive shock to septic shock with continued norepinephrine dependence. He was empirically started on meropenem 1 g daily, linezolid 600 mg twice daily, levofloxacin 750 mg, and fluconazole 100 mg once daily for 48 hours, pending culture data. The pulmonary, critical care, and infectious diseases teams were also consulted regarding critical illness physiology and suspected disseminated tuberculosis.

**Figure 2:**
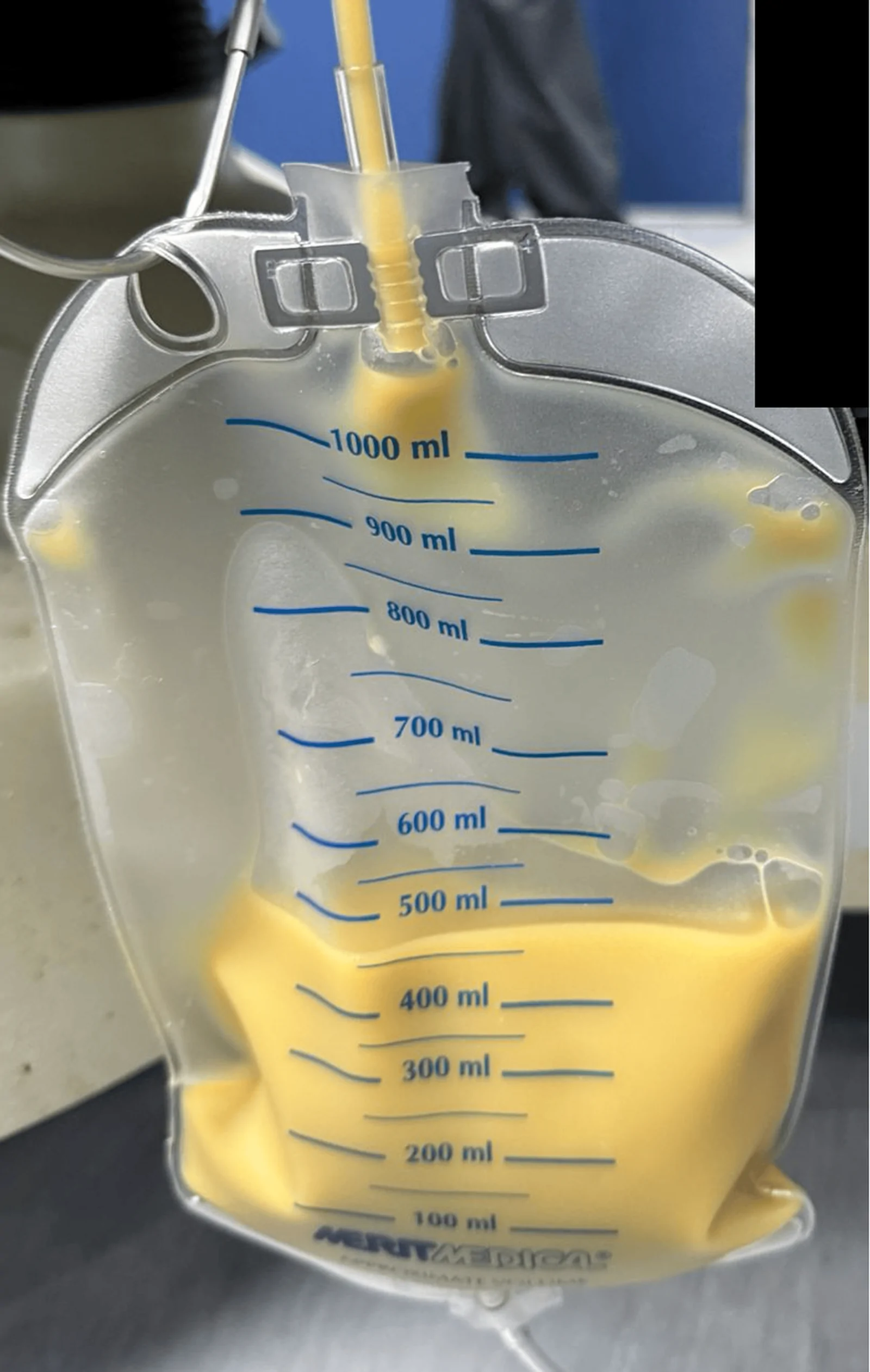
Pericardial aspirate (total of 600 mL) demonstrating the characteristic appearance of purulent fluid collected during emergency pericardiocentesis

The patient was taken to the operating room on the following day, where he underwent right VATS with pericardial window and washout (Figure 3). Copious purulent material was again evacuated from the pericardial sac, and a 32 French intercostal chest tube drain was placed into the pericardial space. Pericardial fluid culture obtained from the index pericardiocentesis isolated *Streptococcus* species, but local laboratory limitations precluded speciation or antimicrobial susceptibility testing. Blood, bronchoalveolar lavage, pleural fluid, sputum, and urine cultures returned no bacterial or fungal growth after three to seven days of incubation. The infectious disease team de-escalated him to ceftriaxone 1 g twice daily. Sputum and pericardial acid-fast bacillus staining and tissue tuberculosis polymerase chain reaction (PCR) were negative, which ruled out active tuberculosis infection. Pleural fluid lipid analysis did not suggest chylothorax.

**Figure 3:**
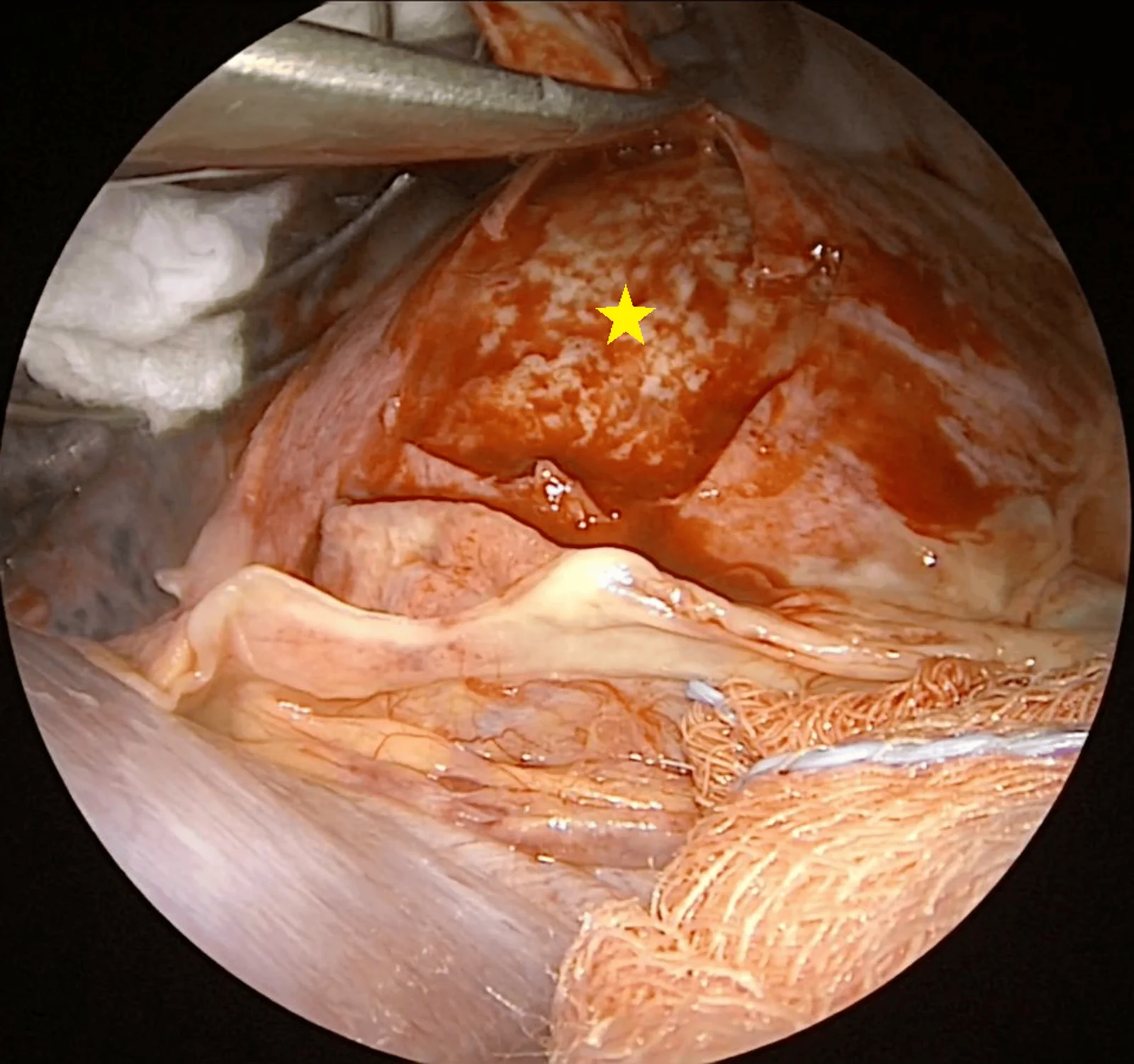
Real-time intraoperative view of the pericardial window and the residual purulent exudate on the visceral pericardium (yellow star)

Inotropic support was gradually weaned, and the patient was extubated on postoperative day 4. The pericardial and intercostal drains were removed the day before because of minimal output. The white cell count fell from 27.5 x 10^9/L over the first four days of admission to 11.8 x 10^9/L by discharge, paralleled by a decline in CRP from 462.9 mg/L on admission to 30-40 mg/L. A follow-up transthoracic echocardiogram two weeks into admission showed a preserved left ventricular ejection fraction of 60-65%, trivial residual pericardial effusion, and a mild left-sided pleural effusion. Histopathological examination of both pericardial and pleural tissue showed no evidence of granulomatous inflammation, and the pericardial findings were consistent with suppurative pericarditis. The patient was discharged 19 days after admission on a three-month course of colchicine 0.5 mg once daily, with planned follow-up in the thoracic surgery, infectious diseases, and cardiology outpatient clinics.

## Discussion

PP is a rare, fulminant pericardial syndrome characterized by macroscopic or microscopic pus in the pericardial cavity, most commonly caused by bacterial pathogens [1,2]. In the pre-antibiotic era, it was a relatively common autopsy finding, complicating up to 40% of pneumococcal pneumonia [3]. In contemporary practice, it accounts for less than 1% of pericardial diseases and fewer than one in 18,000 hospital admissions [4,13]. Notwithstanding this rarity, untreated PP is uniformly fatal, and even with timely intervention, mortality remains approximately 20-40%, principally driven by CT, septic shock, and the late sequelae of constrictive pericarditis, which complicates 20-30% of bacterial pericarditis [5,6,14].

Cardiac tamponade is a pericardial syndrome characterized by compression of the cardiac chambers due to fluid, blood, pus, or air accumulation within the pericardial sac, with resultant impaired diastolic filling, reduced cardiac output, and progression to obstructive shock if not urgently relieved [1,15,16]. Our patient presented with the classic clinical triad of hypotension, jugular venous distension, and muffled heart sounds, along with diffuse concave-upward ST-segment elevation and PR depression characteristic of acute pericarditis [11,17]. Transthoracic echocardiography remains the diagnostic modality of choice, allowing rapid bedside identification of pericardial effusion, semi-quantitative assessment of its volume, and direct visualization of hemodynamic markers of tamponade physiology, namely right atrial systolic collapse, right ventricular diastolic collapse, a plethoric inferior vena cava, and exaggerated respirophasic transvalvular Doppler variation [12,15].

Approximately 40-50% of all cases of PP are caused by Gram-positive cocci, with *Streptococcus pneumoniae* historically representing the single most common organism in contiguous intrathoracic spread, and *Staphylococcus aureus* predominating in hematogenously and postoperatively acquired cases [2,8,9,18,19]. Other implicated organisms include *Streptococcus anginosus* and viridans groups, *Haemophilus influenzae*, anaerobes, *Mycobacterium tuberculosis*, and *Histoplasma capsulatum* [6,20,21]. In our patient, although the local laboratory was unable to perform speciation or susceptibility testing on the *Streptococcus* isolate, taken together, the presence of frank pericardial pus, an immunocompetent adult host, the absence of dental or oropharyngeal foci, and a heavy lifetime tobacco burden are most consistent with *Streptococcus pneumoniae*, which has a well-established organism-specific association with active smoking in invasive disease [22-24].

*Streptococcus pneumoniae* is a lancet-shaped, Gram-positive, encapsulated diplococcus that colonizes the human nasopharynx in 5-10% of healthy adults and 20-40% of healthy children, with invasion contingent upon a dynamic interplay between host immunity and an arsenal of pneumococcal virulence factors, including the polysaccharide capsule, pneumolysin, neuraminidase, pneumococcal surface adhesin A, and choline-binding protein A [25,26]. Pneumolysin, a cholesterol-binding pore-forming cytolysin, mediates epithelial cell apoptosis, disrupts the alveolar-capillary barrier, and amplifies pro-inflammatory cytokine release via Toll-like receptor 4, facilitating bacterial translocation from the nasopharyngeal niche into the lower respiratory tract, the bloodstream, and ultimately sterile body cavities such as the pericardium [25]. Cigarette smoking is consistently identified as one of the strongest modifiable risk factors for invasive pneumococcal disease in the immunocompetent non-elderly adult, and active smokers exhibit a fivefold increased 30-day mortality from pneumococcal community-acquired pneumonia [27,28]. Our patient’s 25 pack-year tobacco exposure thus represents not only a plausible portal of bacterial entry via altered nasopharyngeal colonization dynamics and impaired mucociliary clearance but also an independent adverse prognostic factor.

Pneumococcal pericarditis usually occurs as a complication of pleuropulmonary infection, via contiguous spread from pneumonia or empyema, although hematogenous seeding during pneumococcal bacteremia has also been described [9,29]. Cigarette smoking, being an independent risk factor for invasive pneumococcal disease, may increase susceptibility to these invasive presentations [24]. Although our patient lacked overt lobar consolidation on cross-sectional imaging and reported no antecedent upper respiratory tract symptoms, subclinical or rapidly resolving pneumococcal pneumonia is recognized in cases of invasive pneumococcal disease. The negative blood, sputum, and bronchoalveolar lavage cultures may reflect prior intravenous antibiotic administration. The contemporary literature includes several analogous reports of pneumococcal PP arising from primary respiratory foci, bacteremic septic arthritis, and disseminated pneumococcal sepsis, often in middle-aged adults with active tobacco exposure and progressing to florid tamponade over hours [8,18,30]. Taken together, the most prudent explanation in our case is hematogenous or contiguous pericardial seeding arising from a smoking-related, subclinical pneumococcal respiratory focus.

Pericardial fluid analysis with Gram stain, aerobic and anaerobic culture, acid-fast staining, mycobacterial culture and PCR, and cytology remains the diagnostic cornerstone of PP, as recommended by both the 2025 European Society of Cardiology (ESC) Guidelines for diagnosis and management of pericardial diseases and the 2025 American College of Cardiology (ACC) Expert Consensus [2,19]. Adjunctive serum and pericardial pneumococcal urinary antigen testing, when available, can substantially shorten the diagnostic interval and is endorsed in contemporary case series [9]. The combination of frank pus on pericardiocentesis, pericardial fluid neutrophil predominance, elevated lactate dehydrogenase, low glucose concentration, and culture of *Streptococcus* species was sufficient to establish the diagnosis in our patient.

Management of PP rests on three concurrent pillars: prompt, complete pericardial drainage; intravenous broad-spectrum antimicrobial therapy with subsequent culture-directed de-escalation; and anti-inflammatory therapy to mitigate the risk of constrictive sequelae [2,19]. Case reports underscore this risk, with rapid progression from small effusion to florid tamponade and post-inflammatory constrictive physiology within hours in pneumococcal PP [8]. Although percutaneous pericardiocentesis affords rapid hemodynamic stabilization and is the procedure of choice in the unstable patient, approximately 40% of patients with frank PP ultimately require surgical drainage owing to loculation, dense fibrinous adhesions, or reaccumulation, and surgical pericardiotomy is associated with lower rates of recurrence and post-inflammatory constriction in observational comparisons [31,32]. The 2025 ESC guidelines and 2025 ACC Expert Consensus accordingly recommend a low threshold for surgical pericardial window in PP, with the progression from initial pericardiocentesis to a VATS-guided pericardial window and washout the following day, reflecting this graded approach [2,19].

Empirical antimicrobial coverage should target the most likely pathogens, including methicillin-resistant *Staphylococcus aureus*, *Streptococcus pneumoniae*, and Gram-negative bacilli pending source identification [2]. Our patient’s initial “golden hour” regimen of meropenem, linezolid, levofloxacin, and fluconazole (after cultures were collected) reflects this broad-spectrum strategy when speciation is unavailable and sepsis is undifferentiated [2,33]. Once *Streptococcus pneumoniae* is confirmed and susceptibilities are known, narrower-spectrum coverage with intravenous ceftriaxone or penicillin G for two to four weeks is generally sufficient, as implemented in the critical care sepsis bundle [2]. However, the rising prevalence of penicillin-non-susceptible pneumococcal isolates mandates susceptibility-guided therapy in all but the most resource-limited contexts [34].

Adjunctive colchicine, robustly supported by the Investigation on Colchicine for Acute Pericarditis (ICAP) and Colchicine for Recurrent Pericarditis (CORP-2) randomized trials, halves the rate of recurrent and incessant pericarditis in idiopathic and viral pericarditis [35,36]. Although neither trial enrolled patients with PP, extrapolation of these data, endorsed by the 2025 ACC Expert Consensus and supported by experience of pneumococcal PP successfully managed with pericardiocentesis, targeted antimicrobials, corticosteroids, and a three-month colchicine course without subsequent constrictive sequelae, supports a three-month course of colchicine 0.5 mg once or twice daily following successful source control in PP, both for symptomatic benefit and prevention of post-inflammatory constriction [19,30,35]. Our patient was accordingly discharged on a three-month course of colchicine with surveillance echocardiography.

This case is instructive on several counts. First, it highlights that PP, despite its rarity, should remain firmly within the differential diagnosis of any patient presenting with cardiac tamponade, even in the absence of overt immunocompromise. Our patient was a relatively young man with no biochemical evidence of diabetes mellitus based on random blood glucose and glycated hemoglobin measurements and no evidence of HIV infection. Second, it reinforces tobacco smoking as a powerful and modifiable risk factor for invasive pneumococcal disease in the immunocompetent young adult and provides further evidence-based rationale for smoking cessation counseling and pneumococcal vaccination in this demographic [27]. Finally, the laboratory limitations encountered, the inability to speciate the pericardial *Streptococcus* isolate or perform pneumococcal urinary antigen testing locally, reflect a persistent diagnostic gap in resource-constrained settings of the Caribbean and other low- and middle-income regions and constitute a target for capacity-building investment.

## Conclusions

We describe a 41-year-old immunocompetent Caribbean South Asian man with a heavy tobacco burden who presented with cardiac tamponade secondary to a *Streptococcus* species-associated PPE. His management required emergent pericardiocentesis, a VATS-guided pericardial window with washout, and targeted antimicrobial therapy, with adjunctive colchicine to prevent recurrence and constriction. The take-home message is to maintain a high index of suspicion for PPE as a cause of cardiac tamponade in the immunocompetent host and to recognize tobacco exposure as a modifiable risk factor for invasive pneumococcal disease.
